# Persevering with the Family Health Strategy’s comprehensive community-based approach

**DOI:** 10.1590/0102-311XEN077226

**Published:** 2026-06-22

**Authors:** Ligia Giovanella, Maria Helena Magalhães de Mendonça, Estela Márcia Saraiva Campos, Ana Luiza Queiroz Vilasbôas, Rosana Aquino, Luiz Augusto Facchini

**Affiliations:** 1 Centro de Estudos Estratégicos da Fiocruz Antonio Ivo de Carvalho, Fundação Oswaldo Cruz, Rio de Janeiro, Brasil.; 2 Escola Nacional de Saúde Pública Sergio Arouca, Fundação Oswaldo Cruz, Rio de Janeiro, Brasil.; 3 Faculdade de Medicina, Universidade Federal de Juiz de Fora, Juiz de Fora, Brasil.; 4 Instituto de Saúde Coletiva, Universidade Federal da Bahia, Salvador, Brasil.; 5 Departamento de Medicina Social, Universidade Federal de Pelotas, Pelotas, Brasil.

We would like to thank the seven panelists - experienced managers and researchers - for their comments and reflections on our article ^1^ commemorating the 30th anniversary of the Family Health Strategy (FHS), which provides a critical analysis of its history, institutional and policy milestones and persistent challenges to fully implementing this community-based approach to primary health care (PHC) in the Brazilian Unified National Health System (SUS, acronym in Portuguese). Their comments offer additional insights and draw attention to relevant issues that enrich this debate.

Claunara Schilling Mendonça and Ana Luiza Ferreira Rodrigues Caldas, who served as national managers of PHC in the SUS at different times (as director of the Department of Primary Care and secretary of PHC at the Primary Health Care Secretariat of the Brazilian Ministry pof Health, respectively), highlight the ongoing challenges and concrete opportunities for improving care quality and the PHC model adopted in the SUS ^2^.

The FHS is a unique organizational framework for PHC that has the recognized ability to promote equity and improve health indicators across the life course. However, implementation varies widely, being influenced by geographical, institutional and socioeconomic inequalities. Evidence suggests that settings in which the FHS has a robust structure have a broader scope of services, resulting in high quality care and alignment with the principle of comprehensiveness.

In this regard, in concordance with Mendonça & Caldas ^2^, we emphasize the importance of the provision of technical and financial support by state departments of health, as highlighted by the panelists, especially in small municipalities and remote areas, where the weaknesses of the FHS are most evident. Progress toward even levels of quality requires the adoption of coordinated measures, including a reduction in health team catchment populations, improvements in basic health units (BHU) facilities, the incorporation of digital technologies, the expansion of collaborative practices and the strengthening of continuing education.

Furthermore, as other panelists also point out, we would like to emphasize the importance of ensuring that the workforce is adequately valued by providing stable public sector employment, addressing the currently poor working terms and conditions and ensuring the continuity of health care. The integration of primary and specialized care through matrix support and the expansion of specialized support services is also crucial for managing chronic conditions and enhancing the capacity of PHC to address health needs and deliver effective solutions.

Work process reorganization therefore needs to prioritize interprofessional collaboration, care coordination and health care accountability across territories. Such measures are essential to consolidate the FHS as a cornerstone of health care network organization, thereby reinforcing the principles of universality, comprehensiveness and equity within the SUS.

Dirceu Klitzke, national manager of financing coordination at the Primary Health Care Secretariat, talks about a contemporary PHC agenda and the new guidelines for federal co-financing ^3^, highlighting political and institutional milestones in the development of the FHS, including the expansion of the approach adopted by the strategy. He also reiterates that over the last 30 years national policy has been successful in preserving the integrity of the FHS core team despite varying levels of capacity across municipalities, high workforce turnover and changes in government ^2^.

While federal funding in the form of specific financial transfers allocated to FHS teams has supported the widespread expansion of FHS coverage, national primary care policies (the 2006, 2011 and 2017 Brazilian National Primary Health Care Policy - PNAB, acronym in Portuguese) have revealed tensions between PHC models, as argued by Klitzke ^3^. Notably, the 2017 PNAB reverted to the doctor-centered model, neglecting multidisciplinarity and the territory-based community-oriented approach. While initiatives developed during the current Luiz Inácio Lula da Silva administration have restored priority to the FHS, a new PNAB is urgently needed to compensate the setbacks of the 2017 policy and advance toward the effective implementation and universalization of the FHS care model, which combines quality individual care coordinated across the network and collective disease prevention and health promotion. It is important to enact the PNAB, which, as Houghton & Bascolo ^4^ point out, contributes to the sustainability and consolidation of a policy.

Klitzke ^3^ reiterates the importance of the territorial approach reinstated in the 2024 federal co-financing model and highlights the need to institutionalize practices that address inequities in access to and quality of care stemming from the interaction of structural racism, regional disparities, gender and social class.

Luciano Bezerra Gomes, an experienced researcher and professor at the Federal University of Paraíba (Brazil), highlights the theoretical dispute over care models and reminds the reader of the constant tensions and political wrangling over the implementation of the FHS. He questions the validity of characterizing the implementation of the Family Health Program/FHS as an inflection point for the PHC care model in the SUS ^5^. Indeed, it is not a point of rupture, but rather a process that has been ongoing for the last three decades, marked by advances, setbacks and uneven implementation, as we outline in the article.

One model stands out among the various notions of health care found in the Brazilian literature: that underpinned by the theory of the work and health process. This model entails a structured technological mix designed to solve problems and address individual and collective health needs. A healthcare model is therefore a rationale - a specific logic of organization of health care practices, professionals and services ^6^ - and embodies a significant normative framework. The materialization of this rationale depends on a set of strategic political, institutional and managerial actions aimed at implementing proposals to change hegemonic care models, especially given the complexity of the SUS’s three-tiered governance structure ^6^.

We believe that the FHS is a policy that reorganizes health care practices with a view to upholding the principle of comprehensiveness and eliminating the “symptom-treatment” approach - which underpins private medical-care model - from everyday practice in PHC services. Given the heterogeneity of proposals put forward by different schools of thought drawing on experiments made possible by the SUS decentralization process, in practice the FHS incorporates a variety of alternative proposals for change to care models, such as supportive and welcoming care/expanded care/defense of life, programmatic health actions and health surveillance, which compete with the “symptom-treatment” logic that still persists in many teams ^4^.

Natalia Houghton and Ernesto Bascolo, researchers and technical officers on PHC policy at the Pan American Health Organization (PAHO) in Washington DC (United States), recognize that the FHS in the SUS is a prime example of the construction of a universal system, highlighting the lessons learned and structural requirements shared by health systems for ensuring the effective coordination of care across health networks and consolidating PHC as a strategy ^4^. 

The authors ^4^ highlight that fragmentation of care and poor coordination are major persistent problems and provide an overview of the PAHO’s conceptual and operational framework for Integrated Health Service Delivery Networks (IHSDN), intended to strengthen PHC-based health systems. Indeed, in our article we highlight that care coordination through the integration of PHC across the wider care network is one of the main challenges that needs to be addressed to guarantee the right to access health services.

In full agreement with the authors that care coordination and the configuration of IHSDN should be placed at the forefront of the agenda, we emphasize the requirements for integration: multilevel governance with shared responsibilities and intergovernmental coordination (states and municipalities) with joint management and planning; financing mechanisms that incorporate continuity and network development performance criteria; information infrastructure and telehealth to assess specialized care needs; digital tools that support clinical coordination, territorial service organization and the management and functional integration of networks to ensure the delivery of network-based care ^4^. Furthermore, as Mendonça & Caldas ^2^ also emphasize, digital data integration using electronic health records enables network co-management, organizing care flows starting at the primary care level.

With regard to the SUS, the creation of regionalized health care networks to overcome fragmentation, organizing and scheduling patient flows according to needs, has been a slow process involving the gradual incorporation of shared governance mechanisms. One of the main obstacles is significant private sector involvement in the provision of specialized and hospital care. In addition to a set of IHSDN management mechanisms, a fundamental requirement in Brazil is the expansion of publicly provided specialized services with shared state and municipal management (as in the case of regional polyclinics in Ceará and Bahia). Another requirement is clear and strict regulation of the private sector to ensure compliance with IHSDN guidelines and the principles that underpin the SUS. However, the IHSDN agenda should not be centered on individual care. It is necessary to safeguard the underlying principles of public participation, the integration of health surveillance and timely individual care, disease prevention and health promotion, and the mediation of intersectoral actions to promote health and reduce gender, ethnic/racial and social inequalities.

James Macinko, a researcher at the University of California, Los Angeles (United States) and member of a research group led by Barbara Starfield, celebrates the 30th anniversary of the FHS and its development across thousands of diverse municipalities, highlighting the innovations developed through the strategy’s care model that have created a new vision for community-based PHC and territorial interventions with international repercussions ^7^. 

Macinko ^7^ highlights that the resilience of the FHS is a testament to the ingenuity and perseverance of managers, health workers, researchers and political leaders across Brazil in the work of developing, refining, expanding and improving the strategy, demonstrating dedication and commitment to defending the model and sustaining the FHS in the face of new and emerging threats. 

He recognizes the ongoing challenges and commemorates the FHS after 30 years that have made it the world’s largest comprehensive PHC initiative, urging that it continues to inspire people around the world, coming closer every day to fulfilling its promise of upholding the right to health ^7^.

He urges us to persevere in the work of promoting the effective implementation and universalization of the FHS care model - a territory- and community-based approach to the delivery of quality comprehensive PHC by multidisciplinary teams integrated across the wider health care network and combining individual and collective care in the SUS with social participation.

## Data Availability

Not applicable.
