# Supplementary material for: Persevering with the Family Health Strategy’s comprehensive community-based approach
Source: Cad Saude Publica. 2026 Jun 22;42:e00077226. doi: 10.1590/0102-311XEN077226 (PMC13286396; doi:10.1590/0102-311XEN077226)
Supplement: Translation

# Translation: Perseverar na abordagem integral comunitária da Estratégia Saúde da Família

**DOI:** 10.1590/0102-311XPT077226

**Affiliations:** 1 Centro de Estudos Estratégicos da Fiocruz Antonio Ivo de Carvalho, Fundação Oswaldo Cruz, Rio de Janeiro, Brasil.; 2 Escola Nacional de Saúde Pública Sergio Arouca, Fundação Oswaldo Cruz, Rio de Janeiro, Brasil.; 3 Faculdade de Medicina, Universidade Federal de Juiz de Fora, Juiz de Fora, Brasil.; 4 Instituto de Saúde Coletiva, Universidade Federal da Bahia, Salvador, Brasil.; 5 Departamento de Medicina Social, Universidade Federal de Pelotas, Pelotas, Brasil.

Agradecemos aos sete debatedores, gestores e pesquisadores experientes os comentários e reflexões suscitados por nosso artigo ^1^ que celebra os 30 anos da Estratégia Saúde da Família (ESF) e apresenta uma análise crítica de sua trajetória histórica, marcos políticos institucionais e persistentes desafios para a plena implementação desta abordagem comunitária de atenção primária à saúde (APS) no Sistema Único de Saúde (SUS). Em seus comentários, agregam análises e chamam atenção para questões relevantes que qualificam este debate.

Claunara Schilling Mendonça e Ana Luiza Ferreira Rodrigues Caldas, gestoras nacionais da APS no SUS em distintos momentos (diretora do Departamento de Atenção Básica e secretária da Secretaria de Atenção Primária à Saúde - SAPS, do Ministério da Saúde, respectivamente) enfatizam os desafios persistentes e as possibilidades concretas de aprimoramento da qualidade dos cuidados e do modelo de APS do SUS ^2^.

A ESF constitui um arranjo organizacional original de APS, com reconhecida capacidade de promover equidade e melhorar os indicadores de saúde ao longo do curso da vida. Entretanto, sua implementação apresenta heterogeneidades, condicionadas por desigualdades territoriais, institucionais e socioeconômicas. Evidências indicam que contextos nos quais a ESF se estrutura de forma plena exibem maior completude das ações ofertadas, refletindo maior qualidade da atenção e aproximação ao princípio da integralidade.

Nesse sentido, concordando com Mendonça & Caldas ^2^, destacamos a importância do apoio técnico e financeiro das Secretarias Estaduais de Saúde sinalizado pelas debatedoras, especialmente para municípios de pequeno porte e áreas remotas, onde as fragilidades da ESF são mais evidentes. O avanço rumo a padrões mais homogêneos de qualidade requer a adoção de estratégias articuladas, incluindo a redução da população adscrita por equipe, a qualificação da infraestrutura das unidades básicas de saúde (UBS), a incorporação de tecnologias digitais, a ampliação de práticas colaborativas e o fortalecimento da educação permanente.

Adicionalmente, como destacado também pelos outros debatedores, queremos enfatizar a valorização profissional por meio de carreiras públicas estáveis, que superem os vínculos precários e garantam a continuidade dos cuidados de saúde. A integração com a atenção especializada, mediante o apoio matricial e a ampliação da retaguarda assistencial especializada, mostra-se decisiva para o manejo das condições crônicas e para o aumento da capacidade resolutiva da APS.

Portanto, a reorganização dos processos de trabalho deve priorizar a atuação interprofissional, a coordenação do cuidado e a responsabilização sanitária sobre territórios definidos. Tais medidas são fundamentais para consolidar a liderança da ESF no ordenamento das redes de atenção à saúde, fortalecendo os princípios de universalidade, integralidade e equidade no SUS.

Dirceu Klitzke, coordenador geral do financiamento da SAPS, remete a uma agenda contemporânea para a APS e às novas diretrizes para o cofinanciamento federal ^3^. Reafirma marcos político-institucionais no processo de desenvolvimento com a ampliação da abordagem da ESF e reitera o acerto da política nacional em preservar, nesses 30 anos, a integridade da equipe nuclear da ESF em contexto federativo e capacidades de gestão heterogêneas entre municípios, elevada rotatividade de profissionais e mudanças governamentais ^3^.

A indução federal, com transferências financeiras específicas para as equipes ESF, sustentou a ampla expansão da cobertura da ESF, ao mesmo tempo em que as Políticas Nacionais de Atenção Básica (PNAB 2006, 2011 e 2017) refletiram tensões entre projetos de APS, como bem argumenta Klitzke ^3^. Em especial, a PNAB 2017 retoma um modelo médico centrado, descurando a atuação multiprofissional e a abordagem comunitária e territorial. Urge, portanto, a formulação de uma nova PNAB que supere os retrocessos daquela de 2017, ainda que as iniciativas do atual governo Luiz Inácio Lula da Silva tenham retomado a prioridade para a ESF. Necessitamos de uma nova PNAB que avance na plena universalização e efetiva implementação do modelo assistencial da ESF, que alia cuidado individual de qualidade, coordenado na rede e cuidado coletivo na prevenção e na promoção da saúde. Importante seria uma PNAB transposta em lei que, como lembram Houghton & Bascolo ^4^, contribui para a sustentabilidade e consolidação de uma política.

Klitzke ^3^ reitera a importância da abordagem territorial retomada no novo modelo de cofinanciamento federal 2024 e destaca na agenda contemporânea a necessidade de institucionalizar práticas que enfrentem as iniquidades no acesso e cuidado que atravessam a atuação das equipes ESF, decorrentes da interação do racismo estrutural, heterogeneidades dos territórios, gênero e classe social.

Luciano Bezerra Gomes, pesquisador experiente, professor da Universidade Federal da Paraíba (Brasil), aponta a disputa teórica entre modelos assistenciais e lembra ao leitor as constantes tensões e disputas políticas envolvendo a implementação da ESF ^5^. Questiona a pertinência de denominar a implantação do Programa Saúde da Família/ESF como inflexão do modelo assistencial da APS no SUS ^5^. De fato, não se trata de um ponto de ruptura, mas de um processo que se sustenta por três décadas com avanços, retrocessos e implementação heterogênea, como sintetizamos no artigo. 

Entre as diversas noções de modelos assistenciais presentes na literatura brasileira, destaca-se aquela baseada na teoria do processo de trabalho e saúde: combinações tecnológicas estruturadas para a resolução de problemas e atendimento a necessidades de saúde, de caráter individual e coletivo. Modelo assistencial, portanto, é uma racionalidade, uma certa lógica de organização das práticas, profissionais e serviços de saúde ^6^. Carrega em si uma base normativa relevante. Tal racionalidade para tornar-se concreta depende de um conjunto de ações de natureza político-institucional e gerencial, de caráter estratégico, para a implementação de propostas de mudança dos modelos assistenciais hegemônicos, especialmente diante da complexidade da governança tripartite do SUS ^6^.

Consideramos que a ESF representa uma política de reorganização das práticas de saúde, com vistas a alcançar o princípio da integralidade da atenção no SUS, buscando afastar, do cotidiano da APS brasileira, a lógica da “queixa-conduta”, racionalidade que orienta o modelo médico-assistencial privatista. Diante da heterogeneidade propositiva de diversas correntes de pensamento, à luz de experimentações possibilitadas pelo processo de descentralização do SUS, a ESF incorpora, na prática, diversas propostas alternativas de mudanças dos modelos assistenciais, a exemplo do acolhimento/clínica ampliada/em defesa da vida, ações programáticas em saúde, e vigilância em saúde, que têm disputado com a lógica assistencial do padrão “queixa-conduta” ainda persistente em muitas equipes ^4^.

Natalia Houghton e Ernesto Bascolo, pesquisadores e dirigentes da Unidade de APS da Organização Pan-Americana da Saúde (OPAS), em Washington DC (Estados Unidos), reconhecem a ESF no SUS como caso emblemático de construção de um sistema universal, e destacam lições aprendidas e requisitos estruturais compartilhados por sistemas de saúde para a coordenação do cuidado nas redes de atenção e para a consolidação da APS como estratégia ^4^. 

Reconhecem a fragmentação e a insuficiente coordenação do cuidado como importantes e persistentes problemas, e sintetizam o marco conceitual e operativo para Redes Integradas de Serviços de Saúde (RISS) da OPAS para a consolidação de sistemas de saúde fundamentados na APS. De fato, em nosso artigo sinalizamos a coordenação do cuidado com a integração da APS às redes de atenção, para a garantia do direito ao acesso a serviços de saúde, como um dos principais desafios a superar. 

Em pleno acordo com os autores em colocar a coordenação do cuidado e a conformação de RISS no centro da agenda, salientamos os requisitos apontados para a integração: uma governança multinível com atribuições compartilhadas e coordenação intergovernamental entre estados e municípios com gestão e planejamento compartilhados; mecanismos de financiamento que incorporem critérios de desempenho vinculados à continuidade e à conformação da rede; infraestrutura informacional e telessaúde para qualificar as demandas para a atenção especializada, ferramentas digitais que apoiem a coordenação clínica, organização territorial dos serviços, e gestão e integração funcional das redes, gerando de fato uma atenção em rede coordenada ^4^. Como também ressaltam Mendonça & Caldas ^2^, a integração digital de dados com base no prontuário eletrônico possibilita a cogestão da rede, ordenando fluxos assistenciais desde a APS.

No SUS, a constituição das redes regionalizadas no SUS, para superar a fragmentação, ordenando e regulando fluxos conforme necessidades, tem sido um processo lento, com incorporação gradual de mecanismos de governança compartilhada. Um dos obstáculos centrais está na importante participação privada na oferta de serviços especializados e hospitalares. Ademais de um conjunto de mecanismos de gestão para as RISS, no caso brasileiro, um requisito fundamental é a ampliação da oferta pública de serviços especializados com gestão compartilhada entre estado e municípios (como no exemplo das policlínicas regionais do Ceará e da Bahia). Concomitantemente, outro requisito é uma clara e estrita regulação do setor privado para a sua subordinação às diretrizes das RISS e aos princípios do SUS. A agenda das RISS não pode, contudo, centrar-se no cuidado individual. É necessário resguardar os princípios constitutivos de participação social, integração da vigilância em saúde com cuidado individual oportuno, prevenção, promoção, e mediação de ações intersetoriais para a promoção da saúde e redução de desigualdades de gênero, étnico-raciais e sociais.

James Macinko, pesquisador da Universidade de Los Angeles (Estados Unidos) e colaborador no grupo de pesquisas liderado por Barbara Starfield, celebra os 30 anos da ESF, seu desenvolvimento e difusão pelos milhares de municípios do país, e ressalta as inovações do modelo de cuidados da ESF que levaram a uma nova visão para a atenção primária à saúde de orientação comunitária e intervenções territoriais com repercussões internacionais ^7^. 

Macinko ^7^ considera a resiliência da ESF um testemunho de engenhosidade e perseverança, no trabalho de desenvolver, refinar, expandir e aprimorar a estratégia, de gestores, profissionais de saúde, pesquisadores, líderes políticos de todo o Brasil, com dedicação e compromisso na defesa do modelo e a sustentação da ESF diante de novas e emergentes ameaças.

Reconhece os desafios persistentes e celebra a ESF nos seus 30 anos que a tornaram a mais ampla experiência de APS integral em todo o mundo e almeja que continue a inspirar as pessoas em todos os países, ensejando o direito à saúde ^7^.

Nos convoca a perseverar na efetivação e universalização do modelo assistencial da ESF, uma abordagem de APS integral, de orientação comunitária, territorializada, com equipes multiprofissionais, resolutiva de qualidade, integrada à rede, que alia cuidado individual e coletivo, no SUS com participação social.

## Data Availability

Não se aplica.
